# Zinc-Intercalated Halloysite Nanotubes as Potential Nanocomposite Fertilizers with Targeted Delivery of Micronutrients

**DOI:** 10.3390/ma16206729

**Published:** 2023-10-17

**Authors:** Prokopiy Maximov, Evan Dasi, Natalia Kalinina, Alexey Ruban, Boris Pokidko, Maxim Rudmin

**Affiliations:** 1 Division for Geology, School of Earth Sciences & Engineering, Tomsk Polytechnic University, 634050 Tomsk, Russia; 2 Institute of Geology of Ore Deposits, Petrography, Mineralogy and Geochemistry RAS (IGEM RAS), 119017 Moscow, Russia; 3 Institute of Environmental and Agricultural Biology (X-BIO), University of Tyumen, 625003 Tyumen, Russia

**Keywords:** halloysite, nanotubes, zinc sulfate, nanocomposites, targeted delivery, slow-release fertilizers, chemical activation

## Abstract

This study reports on the development of nanocomposites utilizing a mineral inhibitor and a micronutrient filler. The objective was to produce a slow release fertilizer, with zinc sulfate as the filler and halloysite nanotubes as the inhibitor. The study seeks to chemically activate the intercalation of zinc into the macro-, meso-, and micropores of the halloysite nanotubes to enhance their performance. As a result, we obtained three nanocomposites in zinc sulfate solution with concentrations of 2%, 20%, and 40%, respectively, which we named Hly-7Å-Zn2, Hly-7Å-Zn20, and Hly-7Å-Zn40. We investigated the encapsulation of zinc sulfate in halloysite nanotubes using X-ray diffraction analysis, transmission electron spectroscopy, infrared spectroscopy (FTIR), and scanning electron microscopy with an energy-dispersive spectrometer. No significant changes were observed in the initial mineral parameters when exposed to a zinc solution with a concentration of 2 mol%. It was proven that zinc was weakly intercalated in the micropore space of the halloysite through the increase in its interlayer distance from 7.2 to 7.4. With an increase in the concentration of the reacted solution, the average diameter of the nanotubes increased from 96 nm to 129 nm, indicating that the macropore space of the nanotubes, also known as the “site”, was filled. The activated nanocomposites exhibit a maximum fixed content of adsorbed zinc on the nanotube surface of 1.4 wt%. The TEM images reveal an opaque appearance in the middle section of the nanotubes. S SEM images revealed strong adhesion of halloysite nanotubes to plant tissues. This property guarantees prolonged retention of the fertilizer on the plant surface and its resistance to leaching through irrigation or rainwater. Surface spraying of halloysite nanotubes offers accurate delivery of zinc to plants and prevents soil and groundwater contamination, rendering this fertilizer ecologically sound. The suggested approach of activating halloysite with a zinc solution appears to be a possible route forward, with potential for the production of tailored fertilizers in the days ahead.

## 1. Introduction

Halloysite is a unique mineral (Al_2_Si_2_O_5_(OH)_4_) of the class of phyllosilicates and the kaolinite-serpentine group. Its distinctive feature is the morphology of the particles, which are natural nanotubes [1,2,3,4]. Chemically, halloysite is like kaolinite and has a hollow tubular structure in the submicron range [5,6]. Halloysite can be divided into 10Å-halloysite and 7Å-halloysite, where angstroms show the interlayer spacing of the crystal structure [7]. In a geologic context, halloysite can form in many environments where conditions are present for its formation, including volcanic, tropical, and glacial environments. It is common in many rock types, including volcanic, sedimentary, and hydrothermal rocks [8,9]. The best-known deposits of halloysite are found in New Zealand, the United States [10], France [11], and Turkey [12]. In New Zealand [13], halloysite is mined from rhyolite rocks in the Matauri Bay deposit [14,15]. Halloysite from New Zealand is used mainly in the production of high-quality tableware because of its high whiteness and translucency [16].

Halloysite nanotubes have a micro-needle, roll-like morphology with macro-, meso-, and microporous spaces and high specific surface area, making them efficient containers for the targeted delivery of various agents [17,18,19]. They are low cost. Many studies have investigated halloysite for making polymers with high fire resistance and reduced thermal conductivity [20,21,22,23]. In cosmetics, halloysite can be used as a carrier, such as keratin for hair [24]. In pharmaceuticals, halloysite is used as a filler for various drug and food agents [16,17,25,26,27,28,29,30,31,32,33,34] and for making drugs with antimicrobial properties. For environmental remediation, halloysite is used to produce oil emulsifiers, which are non-toxic and contribute to the population of “Alcanivorax borkumensis” bacteria involved in the cleanup of marine areas from oil contamination [35,36].

Halloysite nanotubes are a readily available and cost-effective raw material for various industries, including agriculture, crop processing, and food processing [37,38,39,40]. Halloysite exhibits diverse polar charges on its basal planes, including macro-, meso-, and micropores, which make it a suitable medium for transporting plant growth regulators and micronutrients. Zinc has been identified as a prospective filler material for nanotubes.

Zinc plays a crucial role in plant nutrition, participating in various physiological and biochemical processes, including photosynthesis and the synthesis of growth hormones [41,42,43]. Zinc deficiency leads to slower plant growth and reduces plant resistance to fungal diseases [44]. Microfertilization with zinc in the form of sulfate depends on the soil’s geochemistry and pH. For instance, zinc becomes unavailable to plants in calcareous soils with high phosphorus and organic matter content [45,46,47]. Furthermore, due to its mobility in wet soils, zinc, changing into ionic form, can leach into groundwater, which subsequently leads to the saturation of water bodies and negatively affects the fauna of the environment [48]. Controlled-release zinc fertilizers can be used to combat these effects [44,49,50,51].

The purpose of the study is to incorporate zinc into macro-, meso- and micropores of halloysite nanotubes by chemical activation to create composites with targeted delivery of trace elements.

## 2. Materials and Methods

### 2.1. Minerals and Materials

Halloysite nanotube concentrate supplied by Halloysite-Ural LLC (Chelyabinsk, Russia) was used as a mineral raw material in this work. Zinc was chosen as the active component to obtain nanocomposites. A zinc sulfate solution with a zinc concentration of 22% was used as a source of nutrient additive.

### 2.2. Chemical and Mechanochemical Preparation of Nanocomposites

The primary stage in the activation of nanocomposites entailed soaking tubular hal-loysite crystals in a zinc sulfate solution. A solution containing zinc was prepared by adding a 22% concentration of zinc sulfate to distilled water. Next, 10 mL of the resulting zinc solution was added to 40 g of halloysite nanotubes. The mixture was thoroughly combined and then dried in petri dishes for 48 h at room temperature. Solutions containing different concentrations of zinc sulfate—2%, 20%, and 40% (zinc concentration 0.4, 4, 8%, respectively)—were utilized in the production of nanocomposite fertilizer. This resulted in the creation of Hly-7Å-Zn2, Hly-7Å-Zn20, and Hly-7Å-Zn40 nanocomposites. Technical abbreviations are defined upon their initial usage.

Prior to activation, the original halloysite sample was dried to eliminate extra moisture from the mineral structure. The pre-treatment procedure entailed drying the original halloysite sample in a desiccator at a temperature of 60 °C for a period of 6 h.

### 2.3. Characterization of the Nanocomposites

In order to investigate the key parameters of the produced nanocomposites and verify the intercalation of zinc into halloysite nanotube structures, a range of laboratory and analytical investigations were conducted which included Fourier transform infrared spec-troscopy (FTIR), laser Raman spectroscopy, X-ray diffraction analysis (XRD), scanning electron microscopy with energy dispersive X-ray spectroscopy (SEM-EDS), and transmission electron microscopy (TEM) with selected area electron diffraction (SAED).

X-ray diffraction analysis was performed to ascertain the bulk mineral composition of the initial halloysite sample and nanocomposites and to gauge the interlayer spacing in halloysite crystals. The analysis was conducted on a Rigaku Ultima IV diffractometer, utilizing a Cu Kα anode at a voltage of 40 kV and a current of 30 mA. Diffraction patterns were obtained in the angle range of 3–65° on a 2θ scale with a rate of 1° per minute and a step of 0.02°, thereby enabling the determination of the crystal structure and interplanar spacing of halloysite.

Scanning electron microscopy was utilized to examine the microstructural characteristics and chemical composition of the nanocomposites. The TESCAN Vega 3 SBU scanning electron microscope (Teskan, Brno, Czech Republic) with an OXFORD X-Max 50 energy dispersive X-ray microanalysis detector (Oxford Instruments, Abingdon, UK) was used for the investigation.

Imaging parameters comprised an accelerating voltage within the range of 10–20 kV, a sample current ranging from 3 to 12 nA, a focal length within the range of 5–15 mm, and operation in full vacuum mode. The analyzed samples were dried crumbly specimens of nanocomposites and starting material. In addition, plant leaves treated with water containing nanocomposites were analyzed. This analysis enabled us to identify the micro-structural characteristics and chemical composition of the nanocomposites, as well as the interaction between plant tissues and halloysite tubes.

Transmission electron microscopy (TEM) was conducted to examine the structure of halloysite nanotubes pre- and post-activation while visually assessing the existence of zinc in the nanotubes’ central region. A JEOL JEM-2100F microscope (JEOL, Tokyo, Japan), with an accelerating voltage of 200 kV, was used for the TEM study. The analytical samples were prepared by converting the ground nanocomposites into a fine powder and then depositing the powder on a copper grid that had been precoated with a carbon film. This technique enabled us to acquire TEM images of the nanocomposites, complemented by local electron diffraction, which further confirmed the structural variations.

IR spectroscopy was utilized to identify chemical bonds and functional groups within the nanocomposites. The spectra were acquired using a Shimadzu FTIR 8400S IR spectrometer in Kyoto, Japan, within the 4000 to 400 cm^−1^ wave number range. The DLATGS detector and KBr pellets provided a resolution of 4 cm^−1^, thereby enabling the analysis of the nanocomposites’ chemical composition and functional groups. Abbreviations and technical terms shall be explained when used for the first time.

Laser Raman spectroscopy was carried out using a Thermo Scientific Fisher DXR2 spectrometer (Thermo Electron Scientific Instruments LLC, Madison, WI, USA) at a laser wavelength of 785 nm and a power of 10–15 mW. Repeated acquisitions were accumulated to improve the signal-to-noise ratio in the spectra with five 10-s scans in the range 0–3300 cm^−1^.

### 2.4. Experimental Methods

To assess the impact of nanocomposites and plants, the flower surface was sprayed with a solution containing Hly-7Å-Zn40 nanocomposite, which was prepared by combining 10 g of the nanocomposite with 0.5 L of distilled water. The resulting mixture was sprayed on the flower from a distance of 10–15 cm from a household sprayer set to fine spraying mode. Also, for comparative analysis, wash-off tests simulating rain were performed by spraying the Hly-7Å-Zn40 nanocomposite and zinc sulfate solution onto pre-moistened plant leaves. Following the application, the plants were kept under normal room temperature conditions for 24 h. After this period, the leaves were cautiously taken off from the plants and scrutinized via scanning electron microscopy (SEM).

## 3. Results

### 3.1. Nanocomposite Morphology

**Figure 1:**
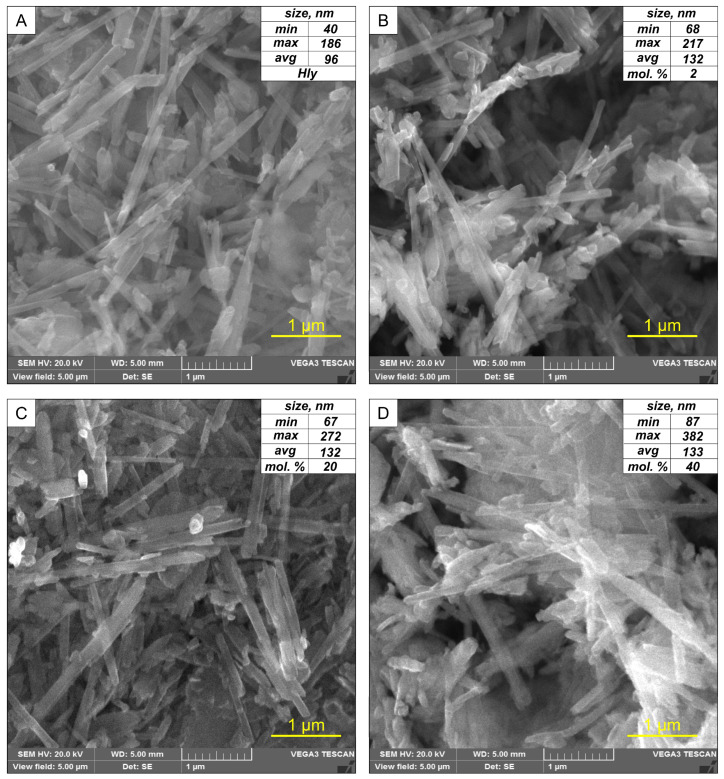
SEM images obtained by secondary electron detector of (**A**) initial halloysite and nanocomposites activated with zinc sulfate solution: (**B**) Hly-7Å-Zn2, (**C**) Hly-7Å-Zn20, (**D**) Hly-7Å-Zn40. The upper-right corner shows the statistical characteristics of nanotube thicknesses.

The initial halloysite concentrate is a concentration of chaotically oriented nanotubes with a length of less than 5 μm and an average diameter of about 96 nm. Morphometric analysis of SEM images was performed to analyze in more detail the morphological changes of the nanotubes after activation. For each nanocomposite, observations were made in 10 sections. The initial width of the tubes averaged between 79 and 114 nm (representing the first and third quartiles). After chemical activation, the increase in the average crystal diameter reaches 127–132 nm (Supplementary Materials, Table S1). The maximum diameter values are observed in the Hly-7Å-Zn40 nanocomposite and reach 382 nm (Figure 1C). These results confirm the morphological changes of the nanotubes after chemical activation.

### 3.2. Structural Characteristics of Nanocomposites

**Figure 2:**
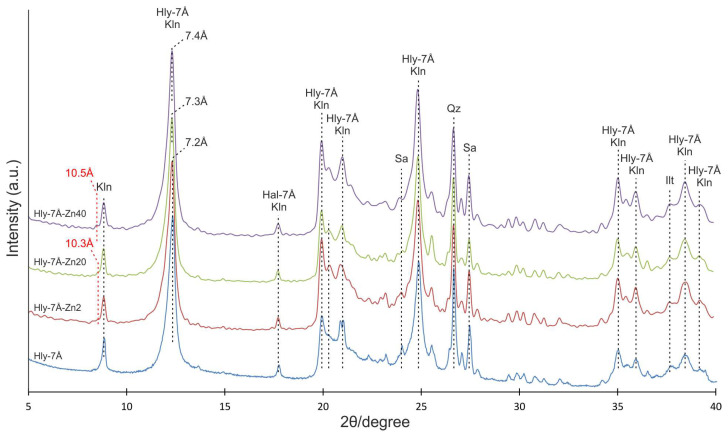
XRD patterns of the initial halloysite concentrate (Hly-7Å) and activated nanocomposites (Hly-7Å-Zn2, Hly-7Å-Zn20, Hly-7Å-Zn40). Hly-7Å—halloysite; Kln—kaolinite; Sa—sanidine; Qz—quartz. The red dotted lines are the new basal peaks (10.3 Å and 10.5 Å).

The X-ray diffraction pattern of the original halloysite indicates the presence of basal reflections characteristic of halloysite, kaolinite, sanidine, and quartz. Basal reflections at 10.0, 7.2, 5.1, 4.5, 4.1, and 3.7 Å correspond to basal reflections of 7 Å and 10 Å-modified halloysite and kaolinite (Figure 2). After activation, the appearance of the first basal reflection (001) at 10.3 Å is observed in the diffractograms of Hly-7Å-Zn2 and Hly-7Å-Zn20 nanocomposites. The Hly-7Å-Zn20 nanocomposite exhibits an increase in basal reflection towards greater interplanar distances, reaching up to 7.3 Å. Meanwhile, the Hly-7Å-Zn40 nanocomposite shows maximum basal reflection shifts at 10.5 and 7.4 Å at the highest concentration of zinc solution (Figure 2).

**Figure 3:**
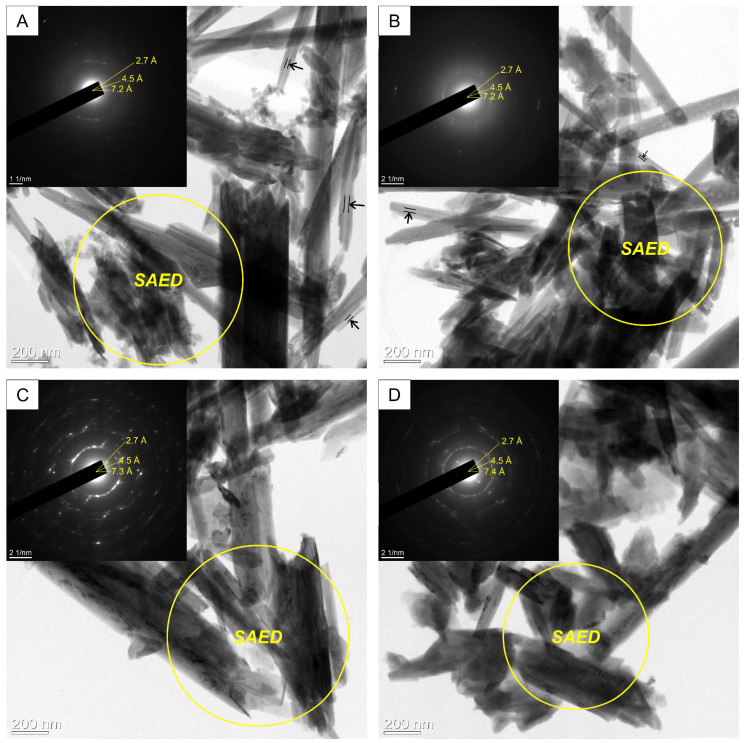
TEM images with local electron diffraction patterns of initial halloysite (**A**) initial and (**B**–**D**) activated by zinc sulfate solution: (**B**) Hly-7Å-Zn2, (**C**) Hly-7Å-Zn20, (**D**) Hly-7Å-Zn40. Note: The arrows show central voids of the initial halloysite and Hly-7Å-Zn2.

In the local electron diffraction (SAED) patterns in the TEM image, the nanotube nanocomposites are characterized by increased interlayer distances relative to the original halloysite (Figure 3). The thickness of the crystal packet from 7.2 to 7.4 Å increases as the solution concentration increases. In the Hly-7Å-Zn2 composite, no increase in the interlayer spacing is observed. An increase in the interlayer spacing is observed in Hly-7Å-Zn20 and Hly-7Å-Zn40 composites. These data agree with the X-ray diffractogram data. TEM images also demonstrate that the central voids in the activated nanotubes (Figure 3B–D) exhibit diminished translucency compared to those of the initial halloysite (Figure 3A).

**Figure 4:**
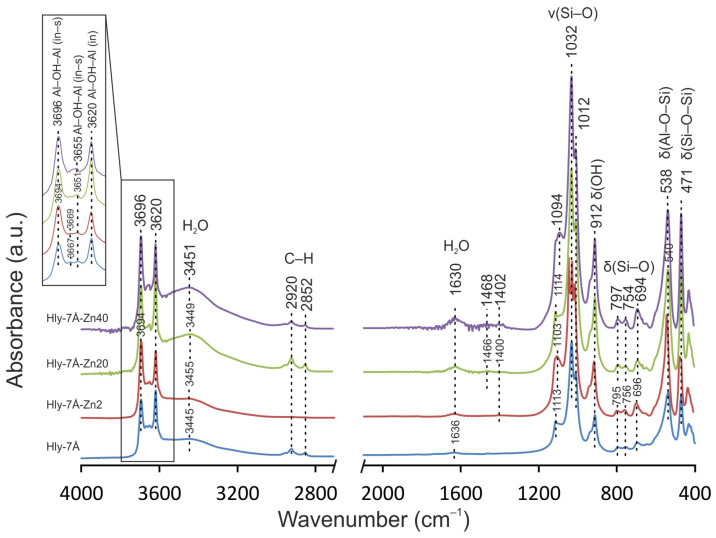
IR spectra of the initial halloysite (Hly-7Å) and activated (Hly-7Å-Zn2, Hly-7Å-Zn20, Hly-7Å-Zn40) nanocomposites.

The infrared spectra of the obtained composites are characterized by strain fluctuations in the range of 3694–3696, 3667–3669, 3651 and 3665 cm^−1^, corresponding to the characteristic groups of the inner surface and inner Al–OH–Al (Figure 4). It is important to note that the strain fluctuations associated with the inner surface OH ions become less pronounced starting from the Hly-7Å-Zn20 composite. It is also observed that the peaks at 3445, 3449, 3451, 3455, 1636, 1630, and 1400–1468 cm^−1^ represent adsorbed water, and an increase in the intensity of the stretching vibrations is observed with increasing solution concentration. At the same time, the absorption bands in the C–H group of the alkylammonium, which are in the range of 3000–2800 cm^−1^, remain almost constant in intensity, as do the symmetric and asymmetric vibrations in the low-frequency range.

**Figure 5:**
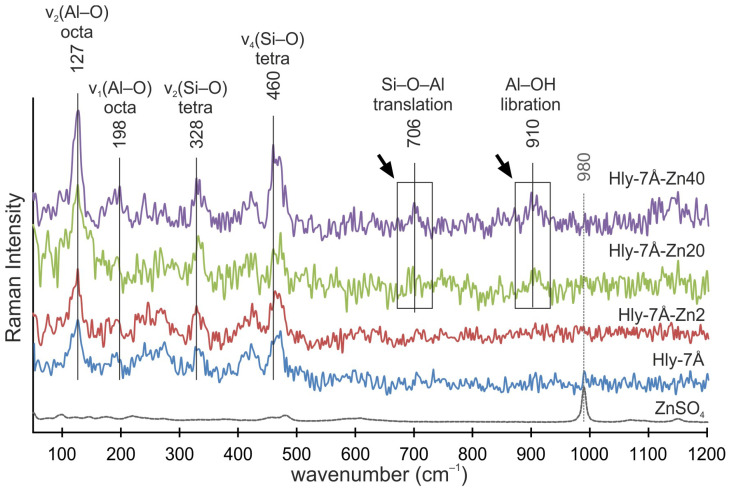
Raman spectra of the ZnSO_4_, initial halloysite (Hly-7Å), and activated (Hly-7Å-Zn2, Hly-7Å-Zn20, Hly-7Å-Zn40) nanocomposites. Black arrows indicate the appearance of new peaks in Hly-7Å-Zn20 and Hly-7Å-Zn40 nanocomposites.

The Raman spectra of the initial halloysite (Hly-7Å) and activated halloysite (Hly-7Å-Zn2) at the minimum concentration are similar (Figure 5). However, Hly-7Å-Zn20 and Hly-7Å-Zn40 showcase new peaks, corresponding to Si–O–Al translation modes at 706 cm^−1^ and the libration mode of the inner Al–OH groups at 910 cm^−1^. The characteristic peaks of zinc sulfate (980 cm^−1^) do not overlap in the spectra of activated nanocomposites (Figure 5). The intensity of the peaks for the 127 cm^−1^ v_2_ (Al–O) and 460 cm^−1^ v_4_ (Si–O) bands increases with increasing zinc concentration in the nanocomposites (Figure 5).

### 3.3. Chemical Composition of Nanocomposites

According to the results of the EDS analysis, the mean constitution of halloysite is as follows: Al_2_O_3_ 42.2–43.8%, SiO_2_ 54.0–55.8%, K_2_O 0.3–1.01%, and Fe_2_O_3_(total) 0.9–1.4% (Supplementary Materials, Table S2). The existence of adsorbed zinc on the surface of the nanotubes in the Hly-7Å-Zn2 and Hly-7Å-Zn20 nanocomposites is not discernible, while zinc signals on the spectra were recorded. The bulk composition analysis of Hly-7Å-Zn40 reveals the presence of zinc within the range of 0.7–1.4 wt.%.

### 3.4. Interaction of Nanotubes with the Plant Surface

**Figure 6:**
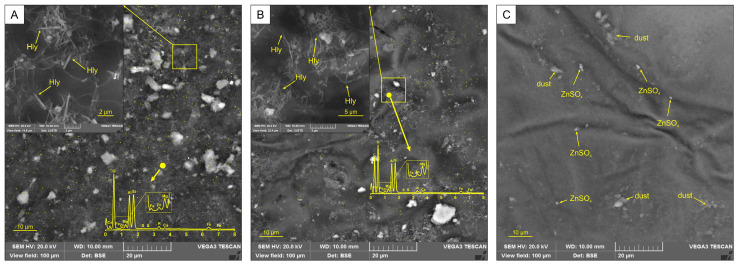
SEM image of nanotubes (**A**,**B**) and map of zinc distribution on the plant tissue surface (**A**–**C**). Yellow points—zinc signals; Hly—halloysite nanotubes.

A detailed study of the interaction of sputtered halloysite and zinc sulfate nanotubes with the surface of plant tissues was carried out using SEM. High-resolution images (Figure 6A,B) clearly show that halloysite nanotubes are attached to the leaf as “needles”. EDS investigation identified zinc signals on the plant surface (Figure 6A,B). After conducting washing tests, a significant number of nanotubes remained on the surface of the plant tissue, and the zinc sulfate had almost wholly disappeared (Figure 6B). The field of view measuring 100 μm shows the even distribution of zinc on the plant’s surface (Figure 6A,B).

## 4. Discussion

Halloysite is not a typical mineral for creating fertilizers. The encapsulation of zinc into halloysite nanotubes using zinc nitrate and borate as a solution [52,53] has been previously discussed to create refractory and anticorrosion coatings or preparations with antimicrobial properties [54]. Halloysite has also been tested as a sorbent for the uptake of zinc and other heavy metals from polluted waters [55,56]. The efficacy of targeting nutrient delivery to plants using biocomposites based on modified halloysite nanotubes has also been reported [57,58]. The positive results seen in the intercalation and adsorption of zinc compounds into halloysite nanotubes, which have demonstrated potential for diverse applications, served as the precursor for this study.

The design of the authors’ work was focused on creating composites of targeted action with the possibility of using them by spraying them on plants, i.e., in the agroindustry. The presence of sharp or fractured edges on halloysite nanotubes facilitates a robust adhesion to plant tissues, enabling a targeted administration of micronutrients (such as zinc) through a “poking” or “injection” mechanism. This feature prevents the undesired removal of these nutrients by rain or irrigation water. The images from the SEM obtained of the plant tissue surface treated with nanocomposite fertilizer demonstrate the observable impact of nanotubular halloysite particles, as shown in (Figure 6A). After washing experiments with rain-simulating water, most of the nanotubes remained attached to the plant tissue surface (Figure 6B). Conversely, the sprayed zinc sulfate was almost entirely removed from the leaf surface (Figure 6C). It was observed that premoistening the leaf surface did not stop the halloysite nanotubes from sticking to it.

The peculiarity of the chemical activation in this work is using zinc sulfate as a solution. According to XRD data in the activated composites, as the concentration of zinc sulfate in the reagent solution increases, a shift of the first basal reflex by about 0.2 Å in the direction of the crystal lattice expansion is observed (Figure 2), which shows the adsorption of zinc in the micropores of the mineral. The expansion of the crystal lattice of halloysite after the activation experiments is also confirmed in TEM images from local electron diffraction patterns (Figure 3). Besides the increase in the interlayer spacing, new basal peaks at 10.3 and 10.5 Å are observed, indicating an increase in the thickness of the 10 Å interlayer spacing of halloysite, which overlapped with the first basal peak of kaolinite (9.95 Å). The last is also confirmed by the increasing intensity of the new basal reflex as the zinc solution concentration increases. At first glance, the interplanar distance increased insignificantly, but according to the morphometry of halloysite nanotubes on SEM images, their width increases by an average of 33 nm as the concentration of the initial reacting solution increases. The linear increase in the average width of the nanotubes shows the expansion of the interlayer distance of the halloysite due to the introduction of the zinc substance into the meso-micro pores (Supplementary Materials, Table S1). Another crucial aspect is that the SEM image (Figure 3A) shows that most of the nanotubes in the original halloysite have a transparent central part. Conversely, this is not the case for the activated composites; the central part of the nanotubes is not transparent (Figure 3B–D). This implies that aside from the interlayer space, the zinc substance also occupies the central part.

During activation, water is used to dissolve zinc sulfate, and it follows that water will inevitably adsorb to the inner and outer surfaces of the crystal structures during activation. However, in the 3400 and 3500 cm^−1^ range, the intensity of vibrations was observed as the concentration of zinc sulfate increased, while the amount of water in the reagent did not change. Thus, the effect of water on the crystal lattice can be ruled out. From this, it can be concluded that the increased vibration intensity associated with adsorbed water is due to the increase in total surface area for aqueous compounds. This conclusion can be related to the increase in interlayer distance. It can also be concluded that zinc ions penetrate more intensively into halloysite structures than water ions. Otherwise, no changes would be seen in the IR spectrometry data. The disappearance of the peak (3667 cm^−1^) responsible for the O-H stretching of internal hydroxides and the formation of an almost monopic peak at 3655 cm^−1^ in Hly-7Å-Zn20 and Hly-7Å-Zn40 nanocomposites are attributed to the hydrolysis of the silinol groups adsorbed on the surface of halloysite nanotubes [52,59,60,61]. This indicates structural modifications of the nanotubes and confirms the activation of halloysite by zinc sulfate. The appearance of new peaks at 706 and 910 cm^−1^ in Raman spectra indicates the intercalation of zinc ions into the halloysite structure. Their intensity also increases with increasing concentration of zinc fraction in the nanocomposites (Figure 5) [62,63]. The absence of characteristic peaks of zinc sulfate in the spectra of the nanocomposites confirms the absence of adsorption of independent forms of zinc sulfate on the surface of halloysite nanotubes.

**Figure 7:**
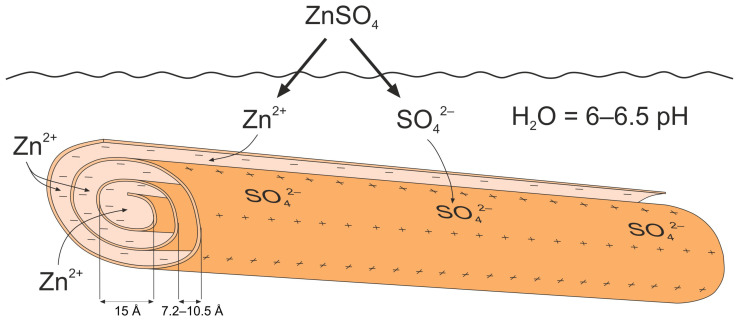
Scheme illustration of the process of zinc incorporation into halloysite nanotube.

According to SEM-EDS data, the amount of zinc adsorbed on the nanotube surface in activated nanocomposites with 2% and 20% zinc sulfate solutions did not stay the same. At the same time, there were intense zinc signals on the spectrum. However, the interlayer distance in the Hly-7Å-Zn20 nanocomposite increased to 7.3 Å, indicating the incorporation of zinc into the mineral structure. The inner and outer walls of halloysite nanotubes exhibit pH-dependent positive and negative charges [34,37]. In acidic environments, the nanotubes’ outer surface develops positive charges due to deprotonation [37]. Conversely, the inner surface acquires positive charges from outer wall protonation in alkaline environments. It should be noted that under highly acidic or alkaline conditions the nanotube structures degrade [64]. The solution was prepared by dissolving zinc sulfate in distilled water with an approximate neutral pH of 5.0–7.0. Under these circumstances, correspondingly, Zn+ cations and SO42- anions from dissolved zinc sulfate will be drawn to the opposing charges of the inner and outer surfaces of the nanotubes (Figure 7). Under this environment, the surface of halloysite nanotubes has a neutral or slightly positive charge (Figure 7) [64]. On the other hand, plant cell membranes are negatively charged, which, according to the laws of electrostatic interactions, favors the penetration into plant tissues and the slow release of positively charged Zn^+^ ions [65]. The presence of a modest positive charge on the surface of the nanotubes may have prevented random adsorption onto the surface. This may explain why the nanotubes took up the whole Hly-7-Zn20 nanocomposite solution, including the large, medium, and small pores. At a 40 mol% zinc sulfate concentration, the Hly-7-Zn40 nanocomposite had 1.4 wt% of zinc on the surface of the nano-needle particles. Also, no independent zinc compounds were observed upon detailed examination of the activated nanotubes via SEM.

The absence of adsorbed zinc in the Hly-7Å-Zn20 nanocomposite increased the interlayer distance. Conversely, in Hly-7Å-Zn40, the unadsorbed forms of zinc sulfate were observed to be fixed on the surface of the nanotube particles. The nanotube macropores exhibit a significant adsorption capacity, enabling them to be filled with most of the solution undergoing the reaction. The different adsorption centers of halloysite, encompassing macro-, meso-, and micro-pores, collectively confer targeted and sustained functionality in nanocomposites.

When using the resulting fertilizer through spraying, zinc release can be adjusted by altering the pH of the sprayed water. As previously mentioned, an alkaline environment leads to deprotonation of the inner surface of the halloysite, resulting in positively charged particles that push the zinc ions outward. This characteristic holds significant importance for agriculture and horticulture. By regulating the pH of the spray water, farmers and gardeners can manage the discharge of zinc from fertilizer into crops. For instance, in alkaline soils, which generally have insufficient zinc levels for plants, the fertilizer can be altered to facilitate the efficient release of zinc in an adequate amount. On the other hand, in acidic soils where zinc solubility is high, zinc release can be reduced to avoid excess accumulation, which could be detrimental to plants and the environment. Therefore, the ability to adjust zinc release based on water pH makes this halloysite and zinc-based fertilizer a controlled tool to optimize plant nutrition and increase yields while minimizing adverse environmental impact.

Despite the many advantages of fertilizers based on halloysite nanotubes, it is essential to consider the potential drawbacks. One of these is that excessive exposure of halloysite to the soil, whether through fertilizer spraying or rainwater runoff, can lead to accumulation of halloysite in the soil.

While halloysite mineral itself does not present any threat to the environment, its existence in soil can have an impact on the chemical equilibrium. Halloysite can increase the pH of the soil, making it more alkaline [66,67]. His alteration in pH can be disadvantageous for certain plant species, particularly those that thrive in acidic conditions. Based on this, it is essential to consider soil and plant characteristics before using halloysite nanocomposites as fertilizer.

## 5. Conclusions

The examination of activated zinc-containing nanocomposites and their comparison with the original zinc-containing nanocomposites resulted in the following conclusions.

(1) The study confirms the potential for zinc intercalation into the meso-microporous spaces of halloysite. It was observed that the minimum concentration of zinc sulfate solution required for this is 20%.
(2) The interaction of halloysite with zinc sulfate is contingent on the concentration of the sulfate solution, affecting both the location and shape of the incorporated zinc within the halloysite structure. Complete absorption of zinc within the nanotube structure is observed upon activation of the halloysite using a 20% zinc sulfate solution. Conversely, when a more concentrated solution (40% zinc sulfate) is used, zinc adsorption in sulfate on the tube surface is observed. This phenomenon suggests a high sulfate concentration, leading to an optimal solution concentration between 0 and 40%.
(3) The intercalation of zinc into the macro-, meso-, and micropores of the halloysite is evident in the subsequent enlargement of the average nanotube diameter and interlayer distance. An increase in the zinc concentration within the solution results in a more substantial increase in the nanotube diameter, thereby signifying a direct correlation between the concentration of infiltrated zinc in the halloysite structure and that in the solution. Furthermore, the successful intercalation is corroborated by PEM data. In the activated nanotubes, the central part of the crystal is opaque, unlike the original halloysite, providing evidence that the tubes are filled with zinc.
(4) Halloysite nanotubes possess a distinctive morphology that enables them to adhere firmly to plant tissues when sprayed on them. These needle-like tubes penetrate and remain on the surface of the leaves, providing a gradual release of zinc and nutrients for the plant. A significant benefit of this technique is that halloysite nanotubes are not washed away by rainwater, unlike fertilizers in the soil.
(5) The surface spraying of halloysite nanotubes permits precise delivery of zinc to plants, avoiding contaminating soil and groundwater, thus rendering the proposed fertilizer environmentally friendly. This technique can play a significant role in sustainable agriculture and environmental preservation.

The study findings suggest that halloysite nanotubes can be used in developing controlled-release zinc fertilizers with low environmental impact. This presents new opportunities for producing efficient and eco-friendly fertilizers for agriculture.

## Data Availability

Data are contained within the article.

## Supplementary Materials

The following supporting information can be downloaded at: https://www.mdpi.com/article/10.3390/ma16206729/s1, Table S1: Nanotube thickness (nm) measurements and statistics; Table S2: Chemical composition of initial halloysite and nanocomposites according to EDS analysis results.

## References

[1] Zahidah K.A., Kakooei S., Ismail M.C., Bothi Raja P. (2017). Halloysite Nanotubes as Nanocontainer for Smart Coating Application: A Review. Prog. Org. Coat..

[2] Joussein E., Petit S., Churchman J., Theng B., Righi D., Delvaux B. (2005). Halloysite Clay Minerals—A Review. Clay Miner..

[3] Kerr P.F. (1952). Formation and Occurrence of Clay Minerals. Clays Clay Miner..

[4] Zhang B., Yu T., Guo H., Chen J., Liu Y., Yuan P. (2022). Effect of the SiO_2_/Al_2_O_3_ Molar Ratio on the Microstructure and Properties of Clay-Based Geopolymers: A Comparative Study of Kaolinite-Based and Halloysite-Based Geopolymers. Clays Clay Miner..

[5] Heidari Pebdani M. (2023). Molecular Insight into Structural and Mechanical Properties of Halloysite Structure. Comput. Mater. Sci..

[6] Theng B.K.G. (1984). Comparison of Intercalation Methods for Differentiating Halloysite from Kaolinite. Clays Clay Miner..

[7] Fukushima K., Kohyama N., Fukami A. (1980). In-Situ Observation of Clay Minerals Hydrated and Intercalated with Liquid Chemicals Using Film-Sealed Environmental Cell. Proc. Annu. Meet. Electron Microsc. Soc. Am..

[8] Merino E. (1989). Aqueous-Chemical Control of the Tetrahedral-Aluminum Content of Quartz, Halloysite, and Other Low-Temperature Silicates. Clays Clay Miner..

[9] Wada K. (1988). Synthesis and Characterization of a Hollow Spherical Form of Monolayer Aluminosilicate. Clays Clay Miner..

[10] Takahashi T., Dahlgren R.A., Theng B.K.G., Whitton J.S., Soma M. (2001). Potassium-Selective, Halloysite-Rich Soils Formed in Volcanic Materials from Northern California. Soil Sci. Soc. Am. J..

[11] Perruchot A., Dupuis C., Brouard E., Nicaise D., Ertus R. (1997). L’halloysite Karstique: Comparaison Des Gisements Types de Wallonie (Belgique) et Du Perigord (France). Clay Miner..

[12] Ece O.I., Nakagawa Z.-E., Schroeder P. (2003). Alteration of Volcanic Rocks and Genesis of Kaolin Deposits in the Sile Region, Northern Istanbul, Turkey. I: Clay Mineralogy. Clays Clay Miner..

[13] Parfitt R.L., Russell M., Orbell G.E. (1983). Weathering Sequence of Soils from Volcanic Ash Involving Allophane and Halloysite, New Zealand. Geoderma.

[14] Bac B., Dung N., Khang L., Hung K., Lam N., An D., Son P., Anh T., Chuong D., Tinh B. (2018). Distribution and Characteristics of Nanotubular Halloysites in the Thach Khoan Area, Phu Tho, Vietnam. Minerals.

[15] Wilson I., Keeling J. (2016). Global Occurrence, Geology and Characteristics of Tubular Halloysite Deposits. Clay Miner..

[16] Mobaraki M., Karnik S., Li Y., Mills D.K. (2021). Therapeutic Applications of Halloysite. Appl. Sci..

[17] Zsirka B., Vágvölgyi V., Horváth E., Juzsakova T., Fónagy O., Szabó-Bárdos E., Kristóf J. (2022). Halloysite-Zinc Oxide Nanocomposites as Potential Photocatalysts. Minerals.

[18] Cheng C., Song W., Zhao Q., Zhang H. (2020). Halloysite Nanotubes in Polymer Science: Purification, Characterization, Modification and Applications. Nanotechnol. Rev..

[19] Zhang Y., Ouyang J., Yang H. (2014). Metal Oxide Nanoparticles Deposited onto Carbon-Coated Halloysite Nanotubes. Appl. Clay Sci..

[20] Lampropoulou P., Papoulis D. (2021). Halloysite in Different Ceramic Products: A Review. Materials.

[21] Prinz Setter O., Segal E. (2020). Halloysite Nanotubes—The Nano-Bio Interface. Nanoscale.

[22] Lapčík L., Sepetcioğlu H., Murtaja Y., Lapčíková B., Vašina M., Ovsík M., Staněk M., Gautam S. (2023). Study of Mechanical Properties of Epoxy/Graphene and Epoxy/Halloysite Nanocomposites. Nanotechnol. Rev..

[23] Finnerty J., Rowe S., Howard T., Connolly S., Doran C., Devine D.M., Gately N.M., Chyzna V., Portela A., Bezerra G.S.N. (2023). Effect of Mechanical Recycling on the Mechanical Properties of PLA-Based Natural Fiber-Reinforced Composites. J. Compos. Sci..

[24] Cavallaro G., Milioto S., Konnova S., Fakhrullina G., Akhatova F., Lazzara G., Fakhrullin R., Lvov Y. (2020). Halloysite/Keratin Nanocomposite for Human Hair Photoprotection Coating. ACS Appl. Mater. Interfaces.

[25] Cavallaro G., Milioto S., Lazzara G. (2020). Halloysite Nanotubes: Interfacial Properties and Applications in Cultural Heritage. Langmuir.

[26] Chen Y., Geever L.M., Killion J.A., Lyons J.G., Higginbotham C.L., Devine D.M. (2017). Halloysite Nanotube Reinforced Polylactic Acid Composite. Polym. Compos..

[27] Jafari S.M. (2017). An Introduction to Nanoencapsulation Techniques for the Food Bioactive Ingredients. Nanoencapsulation of Food Bioactive Ingredients.

[28] Xie M., Huang K., Yang F., Wang R., Han L., Yu H., Ye Z., Wu F. (2020). Chitosan Nanocomposite Films Based on Halloysite Nanotubes Modification for Potential Biomedical Applications. Int. J. Biol. Macromol..

[29] Wei Y., Liang X., Wu H., Cen J., Ji Y. (2021). Efficient Phosphate Removal by Dendrite-like Halloysite-Zinc Oxide Nanocomposites Prepared via Noncovalent Hybridization. Appl. Clay Sci..

[30] De Silva R.T., Pasbakhsh P., Lee S.M., Kit A.Y. (2015). ZnO Deposited/Encapsulated Halloysite–Poly (Lactic Acid) (PLA) Nanocomposites for High Performance Packaging Films with Improved Mechanical and Antimicrobial Properties. Appl. Clay Sci..

[31] Price R.R., Gaber B.P., Lvov Y. (2001). In-Vitro Release Characteristics of Tetracycline HC1, Khellin and Nicotinamide Adenine Dineculeotide from Halloysite; a Cylindrical Mineral. J. Microencapsul..

[32] Shchukin D.G., Sukhorukov G.B., Price R.R., Lvov Y.M. (2005). Halloysite Nanotubes as Biomimetic Nanoreactors. Small.

[33] Sothornvit R. (2019). Nanostructured Materials for Food Packaging Systems: New Functional Properties. Curr. Opin. Food Sci..

[34] Saadat S., Pandey G., Tharmavaram M., Braganza V., Rawtani D. (2020). Nano-Interfacial Decoration of Halloysite Nanotubes for the Development of Antimicrobial Nanocomposites. Adv. Colloid Interface Sci..

[35] Omarova M., Swientoniewski L.T., Tsengam I.K.M., Panchal A., Yu T., Blake D.A., Lvov Y.M., Zhang D., John V. (2018). Engineered Clays as Sustainable Oil Dispersants in the Presence of Model Hydrocarbon Degrading Bacteria: The Role of Bacterial Sequestration and Biofilm Formation. ACS Sustain. Chem. Eng..

[36] Yu T., Swientoniewski L.T., Omarova M., Li M.C., Negulescu I.I., Jiang N., Darvish O.A., Panchal A., Blake D.A., Wu Q. (2019). Investigation of Amphiphilic Polypeptoid-Functionalized Halloysite Nanotubes as Emulsion Stabilizer for Oil Spill Remediation. ACS Appl. Mater. Interfaces.

[37] Wang C., He Z., Liu Y., Zhou C., Jiao J., Li P., Sun D., Lin L., Yang Z. (2020). Chitosan-Modified Halloysite Nanotubes as a Controlled-Release Nanocarrier for Nitrogen Delivery. Appl. Clay Sci..

[38] Jock Churchman G., Pasbakhsh P., Hillier S. (2016). The Rise and Rise of Halloysite. Clay Miner..

[39] Zhang G., Zhou L., Cai D., Wu Z. (2018). Anion-Responsive Carbon Nanosystem for Controlling Selenium Fertilizer Release and Improving Selenium Utilization Efficiency in Vegetables. Carbon N. Y..

[40] Boro U., Moholkar V.S. (2022). Antimicrobial Bionanocomposites of Poly(Lactic Acid)/ZnO Deposited Halloysite Nanotubes for Potential Food Packaging Applications. Mater. Today Commun..

[41] Broadley M.R., White P.J., Hammond J.P., Zelko I., Lux A. (2007). Zinc in Plants. New Phytol..

[42] Sheoran P., Grewal S., Kumari S., Goel S. (2021). Enhancement of Growth and Yield, Leaching Reduction in Triticum Aestivum Using Biogenic Synthesized Zinc Oxide Nanofertilizer. Biocatal. Agric. Biotechnol..

[43] Alloway B.J. (2009). Soil Factors Associated with Zinc Deficiency in Crops and Humans. Environ. Geochem. Health.

[44] Hafeez B., Khanif Y.M., Saleem M. (2013). Role of Zinc in Plant Nutrition—A Review. Am. J. Exp. Agric..

[45] Lakshmi P.V., Singh S.K., Pramanick B., Kumar M., Laik R., Kumari A., Shukla A.K., Abdel Latef A.A.H., Ali O.M., Hossain A. (2021). Long-Term Zinc Fertilization in Calcareous Soils Improves Wheat (*Triticum aestivum* L.) Productivity and Soil Zinc Status in the Rice–Wheat Cropping System. Agronomy.

[46] Brennan R.F., Bolland M.D.A. (2006). Residual Values of Soil-Applied Zinc Fertiliser for Early Vegetative Growth of Six Crop Species. Aust. J. Exp. Agric..

[47] Hussain S., Maqsood M.A., Rengel Z., Aziz T. (2012). Biofortification and Estimated Human Bioavailability of Zinc in Wheat Grains as Influenced by Methods of Zinc Application. Plant Soil.

[48] Krysanov E.Y., Pavlov D.S., Demidova T.B., Dgebuadze Y.Y. (2010). Effect of Nanoparticles on Aquatic Organisms. Biol. Bull..

[49] Dimkpa C.O., Bindraban P.S. (2018). Nanofertilizers: New Products for the Industry?. J. Agric. Food Chem..

[50] Liu R., Lal R. (2015). Potentials of Engineered Nanoparticles as Fertilizers for Increasing Agronomic Productions. Sci. Total Environ..

[51] Kon’kova T.V., Rysev A.P. (2022). Long-Term Zinc-Containing Microfertilizer Based on Bentonite Clay: Manufacture and Properties. Russ. J. Appl. Chem..

[52] Asadi N., Naderi R., Mahdavian M. (2019). Doping of Zinc Cations in Chemically Modified Halloysite Nanotubes to Improve Protection Function of an Epoxy Ester Coating. Corros. Sci..

[53] Fatima Gillani Q., Ahmad F., Ibrahim Abdul Mutlib M., Sri Melor P., Arogundade A. (2016). Synergistic effects of zinc borate and halloysite nanotubes (HNTS) on char morphology and gases emission of epoxy based intumescent fire retardant systems. ARPN J. Eng. Appl. Sci..

[54] Shi X., Nguyen T.A., Suo Z., Liu Y., Avci R. (2009). Effect of Nanoparticles on the Anticorrosion and Mechanical Properties of Epoxy Coating. Surf. Coat. Technol..

[55] Su Z., Zhang H., Gao Y., Huo L., Wu Y., Ba X. (2020). Coumarin-Anchored Halloysite Nanotubes for Highly Selective Detection and Removal of Zn(II). Chem. Eng. J..

[56] Dong Y., Liu Z., Chen L. (2012). Removal of Zn(II) from Aqueous Solution by Natural Halloysite Nanotubes. J. Radioanal. Nucl. Chem..

[57] Makaremi M., Pasbakhsh P., Cavallaro G., Lazzara G., Aw Y.K., Lee S.M., Milioto S. (2017). Effect of Morphology and Size of Halloysite Nanotubes on Functional Pectin Bionanocomposites for Food Packaging Applications. ACS Appl. Mater. Interfaces.

[58] Bertolino V., Cavallaro G., Lazzara G., Milioto S., Parisi F. (2018). Halloysite Nanotubes Sandwiched between Chitosan Layers: Novel Bionanocomposites with Multilayer Structures. New J. Chem..

[59] Liu M., Jia Z., Jia D., Zhou C. (2014). Recent Advance in Research on Halloysite Nanotubes-Polymer Nanocomposite. Prog. Polym. Sci..

[60] Cheng H., Frost R.L., Yang J., Liu Q., He J. (2010). Infrared and Infrared Emission Spectroscopic Study of Typical Chinese Kaolinite and Halloysite. Spectrochim. Acta A Mol. Biomol. Spectrosc..

[61] Szczepanik B., Słomkiewicz P., Garnuszek M., Czech K., Banaś D., Kubala-Kukuś A., Stabrawa I. (2015). The Effect of Chemical Modification on the Physico-Chemical Characteristics of Halloysite: FTIR, XRF, and XRD Studies. J. Mol. Struct..

[62] Theo Kloprogge J., Frost R.L. (1999). Raman Microprobe Spectroscopy of Hydrated Halloysite from a Neogene Cryptokarst from Southern Belgium. J. Raman Spectrosc..

[63] Frost R.L. (1997). Intercalation of Halloysite: A Raman Spectroscopic Study. Clays Clay Miner..

[64] Joo Y., Joo J.H., Jeon Y., Lee S.U., Sohn D. (2013). Opening and Blocking the Inner-Pores of Halloysite. Chem. Commun..

[65] Mahmoudi M., Azadmanesh K., Shokrgozar M.A., Journeay W.S., Laurent S. (2011). Effect of Nanoparticles on the Cell Life Cycle. Chem. Rev..

[66] Massaro M., Noto R., Riela S. (2020). Past, Present and Future Perspectives on Halloysite Clay Minerals. Molecules.

[67] Lisowska A., Filipek-Mazur B., Sołtys J., Niemiec M., Gorczyca O., Bar-Michalczyk D., Komorowska M., Gródek-Szostak Z., Szeląg-Sikora A., Sikora J. (2022). Preparation, Characterization of Granulated Sulfur Fertilizers and Their Effects on a Sandy Soils. Materials.

